# Modified Plasmids and Inverted Terminal Repeats Enhance Adeno-Associated Virus Production and Performance

**DOI:** 10.3390/ijms27177603

**Published:** 2026-08-25

**Authors:** Nicholas Donohue, Alexandra Bogdanovic, James Conheady, Sharon Davin, Niall Barron, Brian Glennon

**Affiliations:** 1 APC Ltd., Building 11, Cherrywood Business Park, Loughlinstown, D18 DH50 Dublin, Ireland; 2 National Institute for Bioprocessing Research and Training, Foster Avenue, Mount Merrion, Blackrock, A94 X099 Dublin, Ireland; 3 School of Chemical and Bioprocess Engineering, University College Dublin, Belfield, D04 V1W8 Dublin, Ireland

**Keywords:** adeno-associated virus, inverted terminal repeat, AAV, ITR, plasmid design, transfection, transduction, gene therapy

## Abstract

Recombinant adeno-associated virus (rAAV) is a preferred vector in gene therapy, although high production costs inhibit widespread adoption. The most common approach for rAAV production involves transfection of HEK293 cells with three plasmids: pTransgene, pRep/Cap and pHelper. Producing sufficient amounts of these plasmids accounts for up to 40% of total batch costs. Initially, this work aimed to increase plasmid yields by replacing the backbones. While this approach increased pHelper yields, pRep/Cap and pTransgene yields were unaffected. A possible reason was identified: pTransgene contains inverted terminal repeat (ITR) sequences that are essential for rAAV production. ITRs have strong secondary structures (including hairpin loops termed B and C arms) that likely interfere with plasmid production. Therefore, targeted deletions were performed within the ITRs. Partial deletions in both the B and C arms of the ITR were most beneficial, as both plasmid yield and transgene expression increased. Importantly, partial deletions did not reduce rAAV yield, as had been previously observed when the B and C arms were fully deleted. In summary, we report a 140% increase in pHelper plasmid production, while the most successful ITR variant increased pTransgene plasmid yields by 57% and transgene expression by 28%, without reducing rAAV yields or transduction efficiency.

## 1. Introduction

Adeno-associated virus (AAV) is a preferred gene delivery system for the treatment of genetic diseases. Currently, there are eight approved AAV-based drugs, with a further 146 undergoing clinical trials [1,2]. Recombinant AAVs (rAAVs) are used to shuttle a therapeutic transgene into the cells of a patient, thereby compensating for a genetic defect. AAV has a broad range of naturally occurring serotypes that can be used to target delivery into most cell types. The low immunogenicity of AAV is a further advantage, although the maximum DNA cargo capacity of 4.7 kb can be a limiting factor in treating certain diseases [3].

rAAVs consist of an icosahedral capsid and a single-stranded DNA genome. The genome contains the therapeutic transgene, flanked by inverted terminal repeats (ITRs). rAAVs are typically produced through triple transfection of human embryonic kidney cells (HEK293). Using cationic reagents (e.g., polyethylenimine, PEI), three separate plasmids are transferred to the HEK293 nucleus. These plasmids (pTransgene, pRep/Cap and pHelper) carry the therapeutic transgene, the AAV replication (*rep*) and capsid (*cap*) genes and adenovirus-derived helper genes, respectively. In rAAV production, the AAV serotype 2-derived ITR and *rep* genes are used in combination with whichever *cap* gene is required [4].

The triple plasmid system increases safety by reducing the likelihood of recombination events that could produce wildtype AAV or rAAV containing oncogenic helper genes [5]. However, producing these plasmids in large quantities is one of the major costs (up to 40%) in rAAV production [6,7,8]. Alternatives to the triple plasmid system include the use of stable AAV producer cell lines or single plasmids that combine all of the AAV-production elements [9,10]. While these systems simplify AAV production, they reduce flexibility (i.e., require more effort to swap transgenes or *cap* genes), and it is unclear how they compare to the triple plasmid system in terms of unwanted recombination or mis-packaging events [11].

Due to their high G/C content and long stretches of inverted repeats, ITRs pose a challenge for plasmid production—for example, specific strains must be used to maintain stability in *E. coli* [12]. Plasmid yields are therefore likely to be reduced by the presence of ITRs. The ITR structure consists of an ‘A’ arm, which contains the Rep-binding element (RBE), as well as ‘B’ and ‘C’ arms. A secondary Rep-binding element, termed RBE’ is present in either the ‘B’ or ‘C’ arms, referred to as ‘Flip’ or ‘Flop’ orientation, respectively. Further elements include a ‘D’ region and a terminal resolution site, *trs*. These sequences are essential for Rep-mediated copying of the transgene and packaging into capsids. Deletions in these regions usually reduce rAAV yields or make vector genome replication impossible [13].

Once transfected, the transgene is copied by host cell DNA polymerase δ and packaged into capsids by Rep. The helper genes manipulate the cell cycle and promote rAAV production [14]. Importantly, rAAVs are not replication-competent. As a result, gene therapy may require high titers of rAAVs (typically in the range of 10^14^–10^16^ genome copies in a single dose) to simultaneously treat as many target cells as possible [15]. However, given that only specific cells that express the target gene need to be transduced, doses for some privileged targets can be lower (e.g., 1.5 × 10^11^ genome copies per dose for the treatment of retinal cells) [16].

Producing the necessary amounts of rAAV is expensive, and this is reflected in rAAV drug prices. For example, a single dose of Hemgenix for the treatment of hemophilia B has a price of 3.5 million USD [17]. Repeated treatments may also be required, as cell division and apoptosis lead to a gradual loss of the therapeutic transgene [14]. Consequently, there is a strong incentive to improve rAAV production and bring down costs. In this work, we sought to improve the design of rAAV plasmids by minimizing the size of backbone elements, the use of different origins of replication, and targeted modifications of the ITR sequence.

## 2. Results

### 2.1. Plasmid Backbone Minimization

rAAV plasmids contain backbone elements that are necessary but do not contribute to rAAV production. These include elements necessary for plasmid maintenance and selection (origins of replication and antibiotic resistance markers) as well as superfluous DNA (e.g., f1 phage origins of replication).

Transfection of DNA into HEK293 cells has an upper limit as more DNA requires the use of more transfection reagent (e.g., PEI). However, PEI is cytotoxic beyond a certain concentration (4.5 mg/L) [18,19]. Therefore, it is sensible to maximize the amount of rAAV-relevant DNA in the transfection complex.

**Figure 1:**
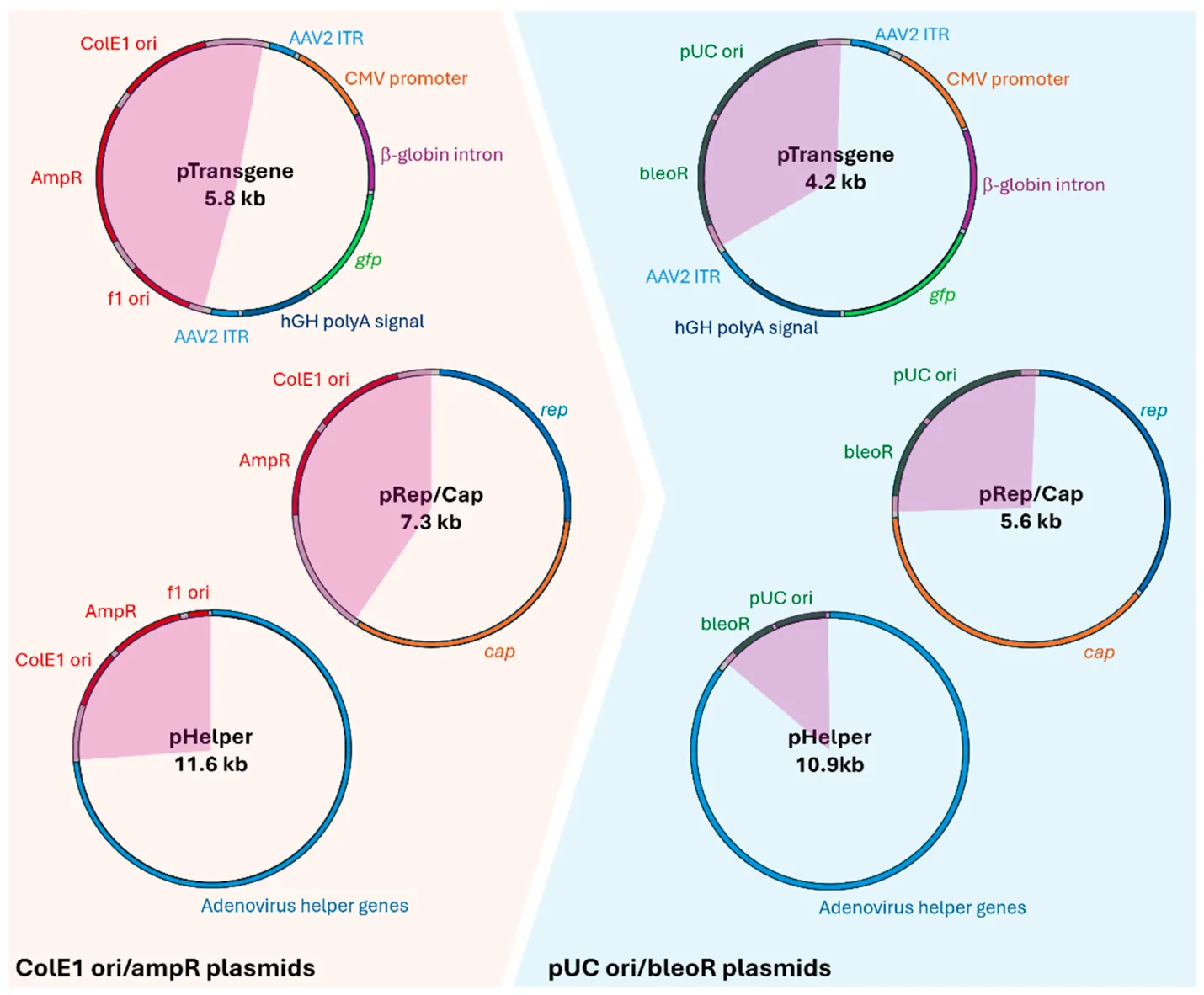
Plasmid backbone modifications. Plasmid names, size in kb and key elements are annotated. Plasmid segments not involved in rAAV production are highlighted in purple. Original ColE1 ori/ampR plasmids are on the left, modified pUC ori/bleoR plasmids are on the right. Plasmids elements drawn to scale.

The initial aim of this work was to reduce the size (in bp) of these backbone elements and eliminate superfluous DNA. Initially, the ColE1 origin of replication was changed to a pUC origin of replication, which is reported to have a higher copy number [20]. The ampicillin resistance marker was changed to bleomycin resistance, as the latter gene is smaller (966 bp vs. 375 bp). Other elements such as the f1 phage origin of replication or non-functional DNA were removed entirely. As a result, the cumulative size of plasmid DNA was reduced by 16%, from 24.7 kb to 20.7 kb (Figure 1) and the proportion of non-rAAV related elements within the plasmids was reduced from 50% to 32% (pTransgene), from 42% to 24% (pRep/Cap and from 18% to 12% (pHelper).

**Figure 2:**
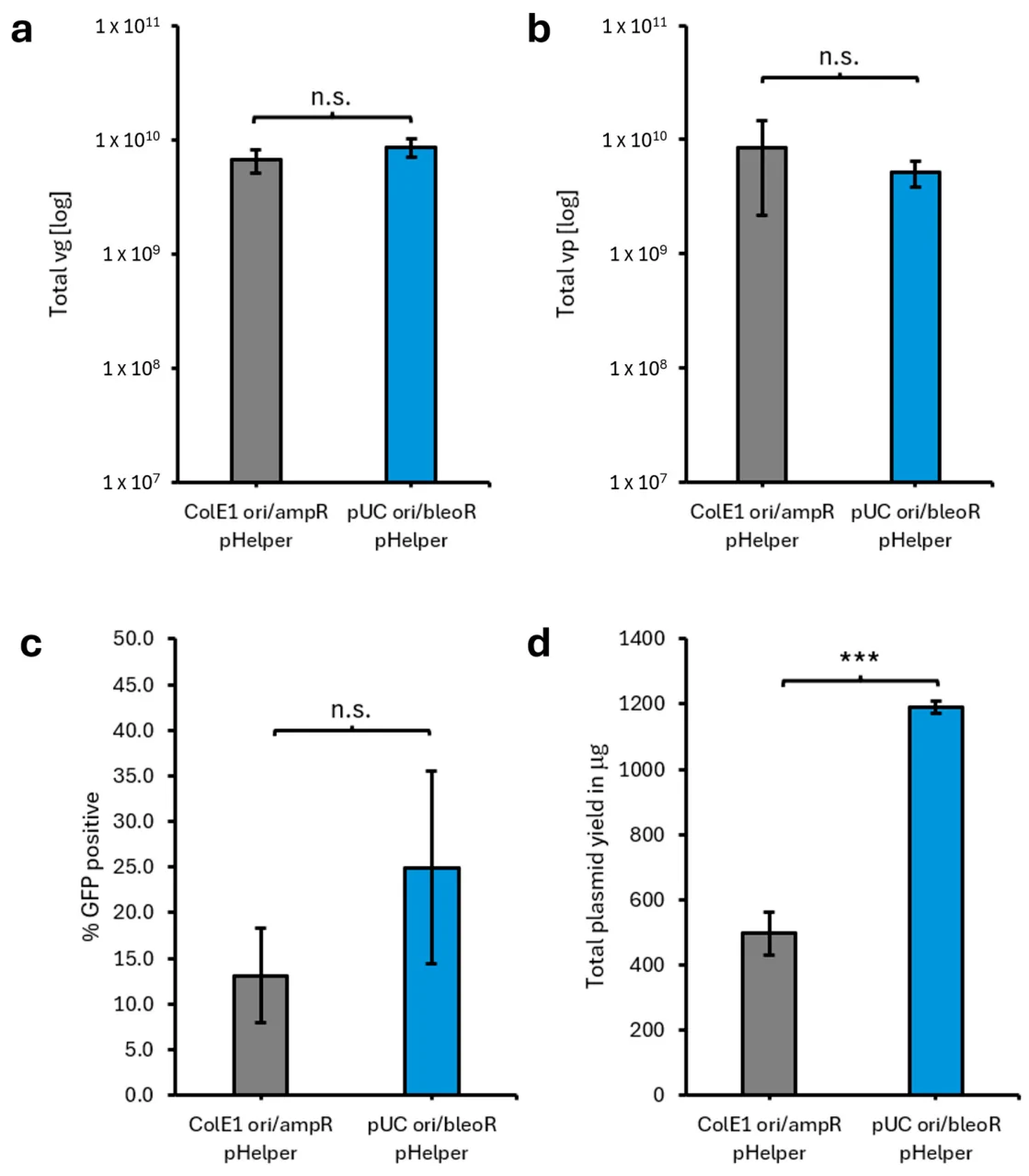
Comparison of original (ColE1 ori/ampR) and pUC ori/bleoR rAAV plasmids. (**a**) Total vector genome (vg) yield (produced from 500 μL HEK293 culture) determined by qPCR; (**b**) total rAAV5 capsid (vp) yield (produced from 500 μL HEK293 culture) determined by ELISA; (**c**) transduction data: CHO cells were transduced at an m.o.i. of 10,000 vg/cell and incubated for 72 h before measuring the percentage of GFP-positive cells by flow cytometry; (**d**) total maxiprep yield of helper plasmid determined by nanodrop. Bars represent average of 6 biological replicates (**a**,**b**) or 3 biological replicates (**c**,**d**). Error bars represent standard deviation. ***, *p* < 0.001; n.s., not significant (*t*-test).

HEK293F cells were triple-transfected with the modified plasmids at a 1:1:1 molar ratio, followed by rAAV purification at 72 hrs. All experiments used rAAV5 as the capsid serotype, unless otherwise indicated, as this serotype is commonly used in currently available rAAV drugs [21]. Although a 29% increase in vector genome (vg) yield was observed by qPCR using pUC ori/bleoR plasmids, this result was not significant (*p* = 0.085, *t*-test) (Figure 2a). Similarly, for capsid yields (vp, determined by ELISA), an increase of 83% was observed using pUC ori/bleoR plasmids, although this result was also not significant (*p* = 0.06, *t*-test) (Figure 2b). These results indicate that the plasmid modifications had a minimal effect on either vector genome or capsid yields.

To ensure the plasmid modifications did not impact the activity of the rAAVs produced, CHO cells were transduced with rAAV5 produced using pUC ori/bleoR or the original plasmids at a multiplicity of infection (m.o.i.) of 10,000 vg/cell. CHO cells were used for these experiments as previous work had indicated that this cell line was best suited for rAAV5 transduction [22]. At 72 h post-transduction, GFP positive cells were measured by flow cytometry. No significant differences were observed (*p* = 0.15, *t*-test). Given that the plasmid modifications did not alter the vector genome element itself, this result aligned with expectations (Figure 2c).

### 2.2. pUC ori/bleoR Plasmid Yields

It was hypothesized that replacing the ColE1 origin of replication with the higher-copy number pUC ori would lead to increased plasmid yields. This was not the case for pTransgene and pRep/Cap, although pUC ori/bleoR pHelper showed significant increases in plasmid yield from miniprep compared to the original pHelper (*p* < 0.05, *t*-test) (Supplementary Figure S1). This result was confirmed by maxipreps in which the pUC ori/bleoR pHelper showed a 140% increase in plasmid yield compared to the original pHelper (*p* < 0.001, *t*-test) (Figure 2d). A new stock of competent *E. coli* was also re-transformed with pUC ori/bleoR pHelper to eliminate the possibility of strain-specific mutations causing an increase in yield. However, the increase in yield was maintained in the re-transformed sample (*p* < 0.001, *t*-test) (Supplementary Figure S1).

In a control experiment, only the ColE1 ori was replaced with pUC ori in the original ColE1 ori/ampR pHelper (i.e., leaving ampR intact) to determine whether the increase in plasmid yield was purely caused by the change in ori. Miniprep experiments showed a significant 14% increase in yield of the pUCori/ampR pHelper compared to the ColE1 ori/ampR pHelper (Supplementary Figure S2a, *p* < 0.05, *t*-test), which indicated that the increase was at least in part caused by the pUC ori replacement.

### 2.3. ITR Modifications

pTransgene may have been unaffected by the insertion of pUC ori due to strong secondary structures in other elements; in particular the high G/C content and long inverted repeats of ITRs in pTransgene are associated with low plasmid stability in *E. coli* [6,7,23]. It is unclear why pRep/Cap yields were unaffected by the insertion of pUC ori, although a region downstream of the *rep* start codon also contains strong secondary structures. This region has been reported to act as an alternative Rep-binding site to initiate replication; given that it performs a similar function to the ITR, it may also inhibit higher plasmid yields [24,25]. Conversely, insertion of a single ITR 208 bp downstream of the pUC ori in the pUC ori/bleoR pHelper plasmid did not significantly reduce yields compared to the same plasmid without ITR insertion (*p* > 0.05, *t*-test; Supplementary Figure S1). Double ITR insertion may be necessary to observe detrimental effects on pHelper plasmid yield.

**Figure 3:**
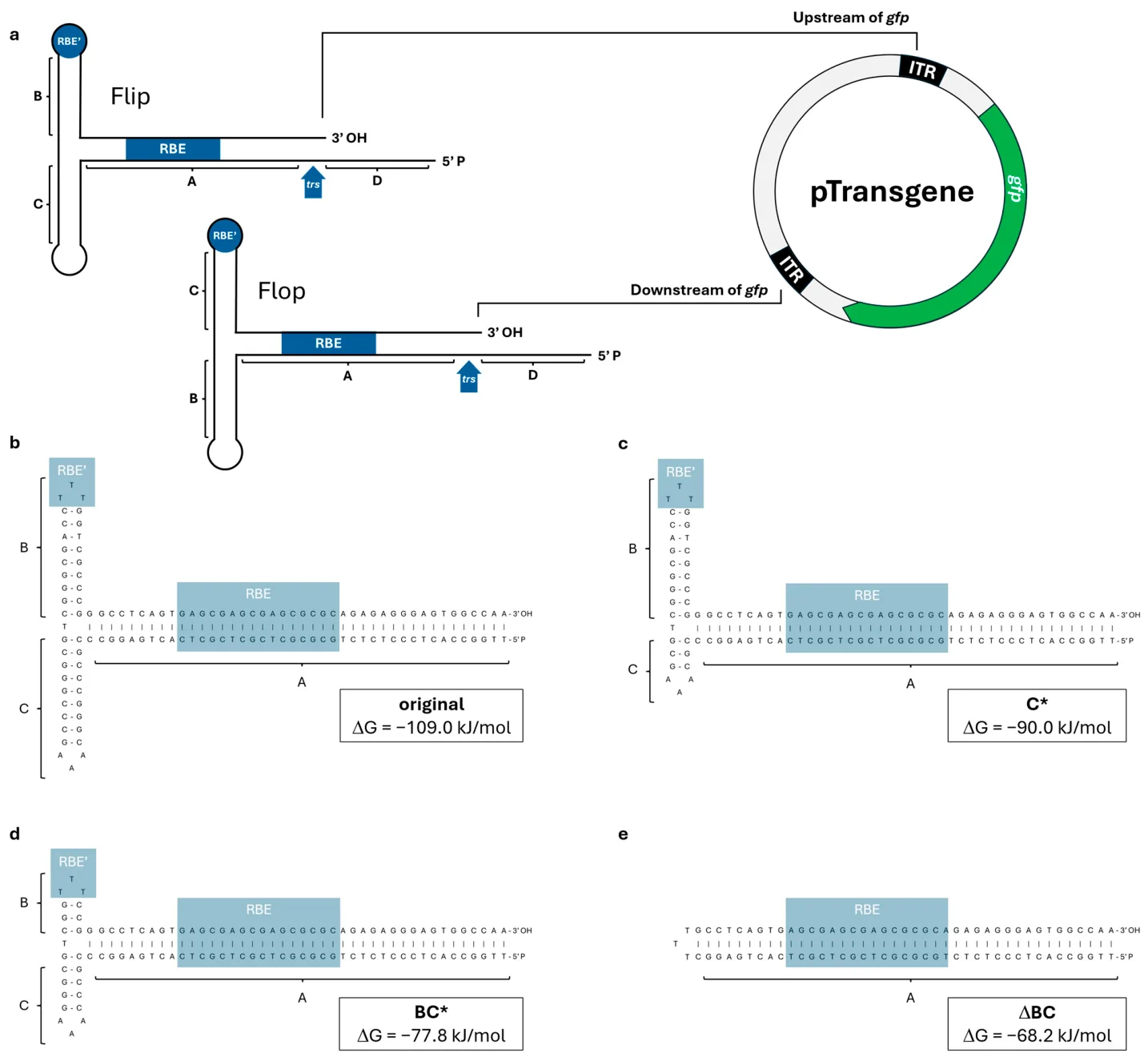
Diagrams and detailed sequences of the original ITR and modified variants. (**a**) Diagram of the ITR structure (Flip and Flop orientation) and positions within the pTransgene plasmid. A, B and C arms are indicated, as well as the D region, primary and secondary Rep-binding elements (RBE, RBE’) and terminal resolution site (*trs*). Structure based on [29]. (**b**–**e**): Detailed sequences and predicted secondary structure of ITR variants (in Flip orientation). (**b**) Original AAV2-derived ITR. (**c**) C* variant. (**d**) BC* variant. (**e**) ΔBC variant. The primary and secondary Rep-binding elements are highlighted (RBE, RBE’ respectively) along with position of the A, B and C arms. The Gibbs minimum free energy (ΔG, kJ/mol) is indicated for each variant. Secondary structure was predicted using software provided by [30].

Given the vital role of ITRs in rAAV genome replication, packaging and transduction, we sought to perform targeted modifications to reduce the strength of the secondary structure. Ideally this would increase plasmid yield while maintaining rAAV yields. The starting material used in this study (i.e., pTransgene, acquired from Takara) contained shortened versions (130 bp) of the wildtype AAV2 ITR (145 bp) flanking the *gfp* gene. Targeted deletions were performed in the ITR structures of pUC ori/bleoR pTransgene to sequentially reduce the overall strength (expressed as Gibbs free energy ΔG, measured in kJ/mol). Initially, 12 bp were deleted in the C arm, which unlike the B arm is not reported to have a secondary Rep-binding element (termed C*; Figure 3c).

In a subsequent iteration, a further 12 bp were deleted in the B arm, leaving both the B and C arms shortened (BC*; Figure 3d). The C* and BC* variants contain only partial deletions and maintain the overall T-shaped structure of the ITR, as opposed to full deletions of the B and C arms explored in other work [26].

In a final variant, both B and C arms were completely deleted, as performed previously [27] (ΔBC; Figure 3e): when both ITRs were replaced with the ΔBC variant, increased transgene expression was observed, but vector genome yields were reduced. Our study sought to replicate these results for comparison as well as test how single ΔBC ITR substitutions performed. The A arm and *trs* were not targeted for deletion, as these structures were considered too important for rAAV production [28] and contained binding sites for the qPCR primers used to quantify vector genomes.

The above ITR variants were inserted either upstream or downstream of *gfp* or in both locations simultaneously in pUC ori/bleoR pTransgene plasmids (Figure 1 and Figure 3a for plasmid maps). Complete ITR sequences are given in Supplementary Table S1. If only a single ITR was changed, the other ITR remained as the original version. All constructs were verified by nanopore sequencing.

### 2.4. ITR Variant Plasmid Yields

Plasmid yields of ITR variant pTransgene supported the initial hypothesis; i.e., weaker secondary structures led to higher plasmid yields (Table 1) compared to the original control, under specific conditions. When the BC* and ΔBC ITR variants were placed exclusively upstream of *gfp* in pTransgene, significantly increased yields of 20% (*p* < 0.05, *t*-test) and 16% (*p* < 0.01, *t*-test), respectively, were observed. The C* ITR variant in this location showed no significant changes. The result for the BC* upstream of the *gfp* variant was confirmed by maxiprep, as this variant showed other beneficial features in later experiments. In this case, a 57% increase compared to the original was observed (*p* < 0.001, *t*-test) (Supplementary Figure S2b).

The effect of ITR variants on plasmid yield also appeared to be cumulative, in that replacing both ITRs with a particular variant caused a stronger effect than a single ITR substitution (Table 1); both the BC* and ΔBC variants showed a 35% increase in yield (*p* < 0.01, *t*-test), while the C* variant showed a 12% increase (*p* < 0.05, *t*-test). Interestingly, no significant changes were observed when the ITR variants were placed exclusively downstream of *gfp* (Table 1).

### 2.5. ITR Variant Vector Genome and Capsid Yields

**Figure 4:**
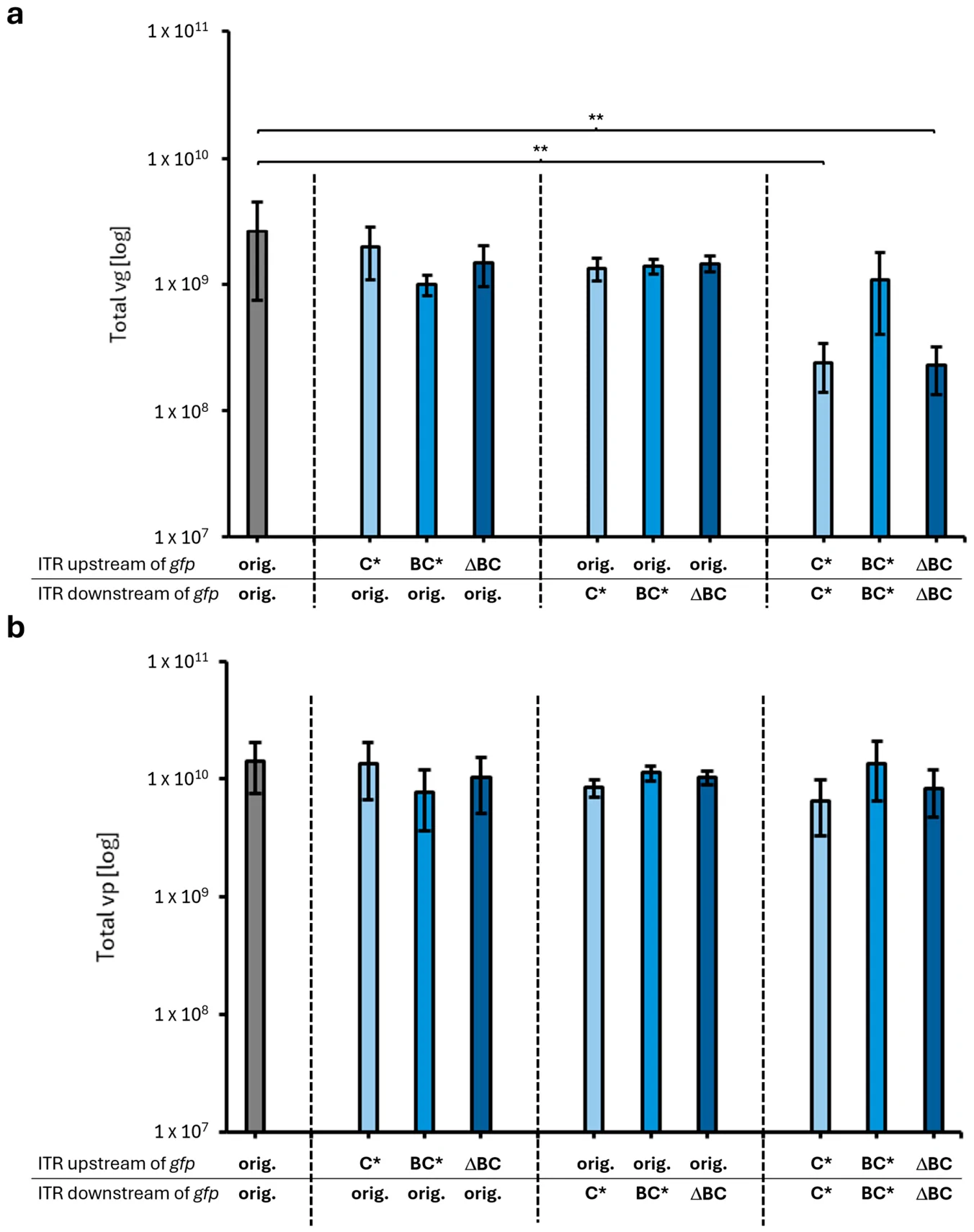
Genome and capsid titers for rAAV5 (produced from 500 μL HEK293 culture) using original (‘orig.’) and ITR-variant plasmids (all with pUC ori/bleoR backbones). (**a**) Total vector genome (vg) yield determined by qPCR; (**b**) total capsid (vp) yield determined by ELISA (no significant differences found). Bars represent average of 6 biological replicates (**a**) or 4 biological replicates (**b**). Error bars represent standard deviation. **, *p* < 0.01 (*t*-test).

In terms of rAAV production, the majority of ITR variants did not significantly affect vector genome yields as measured by qPCR (Figure 4a). However, when the C* and ΔBC variants were inserted in both ITRs, 91% reductions in yield were observed (*p* < 0.01, *t*-test). In contrast, ELISA results indicated that capsid yields were unaffected by any of the ITR variants, regardless of configuration (Figure 4b).

### 2.6. ITR Variant Transduction

**Figure 5:**
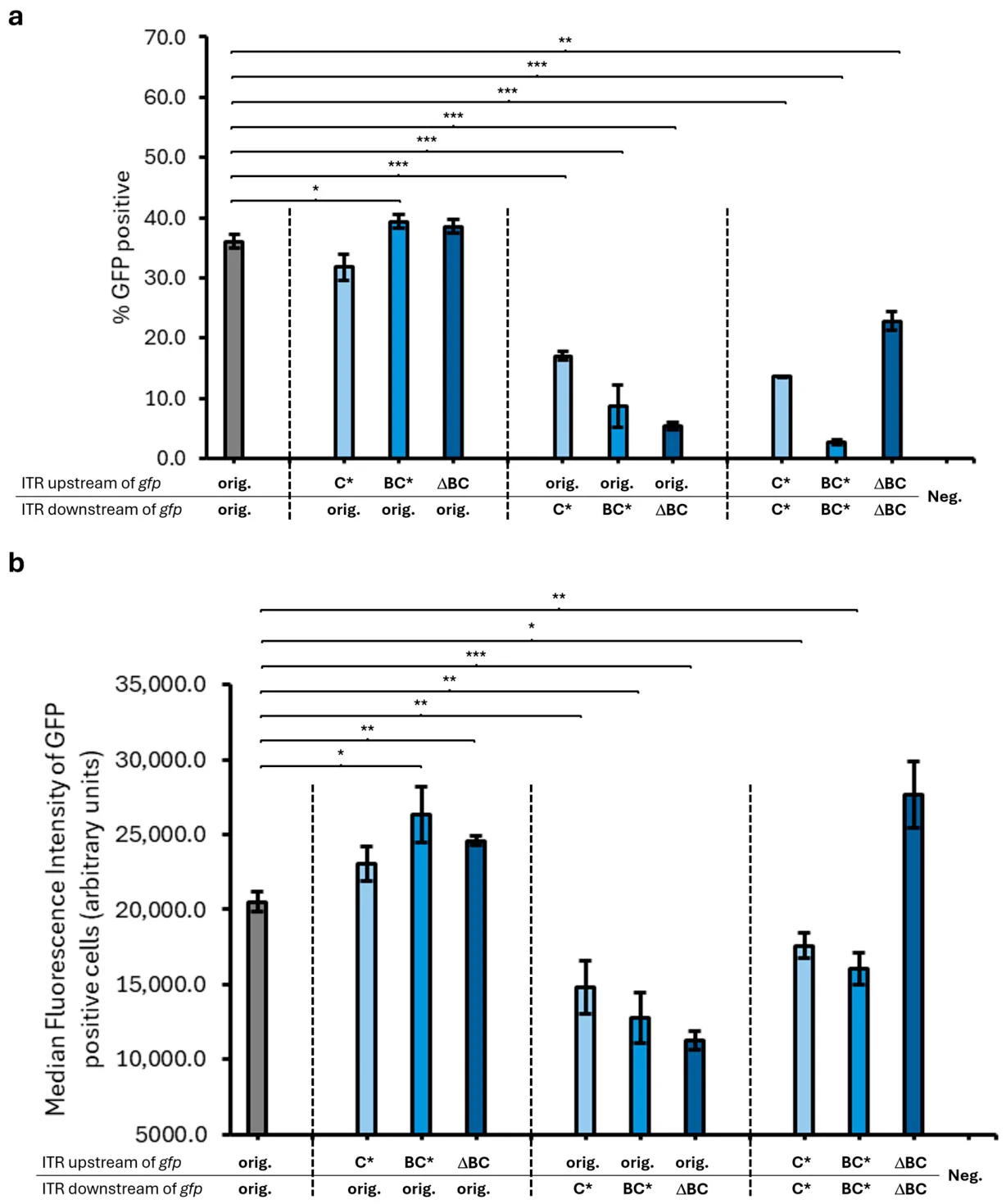
Transduction of CHO cells using original (‘orig.’) and ITR-variant rAAV5 at m.o.i. of 10,000 vg/cell. Flow cytometry was performed at 72 h post-transduction. (**a**) Percentage of GFP positive cells. (**b**) Median fluorescence intensity of GFP positive cells. Bars represent average of 3 biological replicates. Error bars represent standard deviation. Neg., negative control (cells treated with non-AAV control sample). *, *p* < 0.05; **, *p* < 0.01; ***, *p* < 0.001 (*t*-test).

To investigate the transduction ability of ITR-variant rAAVs, CHO cells were transduced at an m.o.i. of 10,000 vg/cell. Compared to original samples, BC* and ΔBC ITRs placed upstream of *gfp* showed significant increases in GFP expression (28% and 20%, *p* < 0.05 and *p* < 0.01, respectively, *t*-test; Figure 5b). The BC* variant in the same configuration also showed a 9% increase in the percentage of GFP positive cells (*p* < 0.05, *t*-test; Figure 5a). However, all variants placed downstream of *gfp* showed a significant reduction in GFP expression and % of GFP-positive (GFP+) cells. Finally, when both ITRs were replaced, C* and BC* variants still showed significant reductions in GFP expression and % of GFP+ cells. On the other hand, while the ΔBC variant showed a significantly reduced % of GFP+ cells, GFP expression was unchanged compared to the original. Collectively, these results indicate that the type of ITR variant and its position relative to *gfp* have a major influence on transduction. This could be related to stability and persistence of the transgene in target cells or effects on *gfp* transcription, or a combination of both.

### 2.7. BC* ITR-Variant Transduction Time Course

Further work aimed to understand how ITR variants affect transduction. A critical step is for the single-stranded rAAV genome to synthesize a second strand and form episomes [31]. This process allows the rAAV genome to persist in the target cell nucleus and express the transgene. We therefore decided to measure transduction over several timepoints (24, 48 and 72 h) to test for delays in GFP expression. This work focused on the BC* ITR variant—when placed upstream of *gfp*, this variant had shown the best overall performance (+57% plasmid yield, unchanged vg and vp yields, +28% transgene expression). For comparison, the BC* ITR variant placed downstream of *gfp* was also included.

On average, the BC* ITR variant placed upstream of *gfp* showed higher plasmid yield and higher median fluorescence intensity following transduction compared to the ΔBC ITR variant placed upstream of *gfp*. Although these differences were not significant (*p* > 0.05, *t*-test), the BC* ITR was considered preferable for further work due to better performance and fewer sequence changes compared to the original ITR.

It was necessary to verify that the BC* ITR variant was indeed present in AAV samples instead of reverting to the original sequence during transfection in HEK293 cells as described previously [32]. A qPCR experiment was performed on AAV samples containing the BC* ITR upstream of *gfp* using primers specific to the BC* ITR variant. Results showed that while the BC* ITR was present within these AAV samples, the titer was significantly lower compared to the original ITR (*p* < 0.001, *t*-test; Supplementary Figure S3). Some of this discrepancy can be explained by the fact that in this variant, only one of the ITRs was replaced with the BC* ITR variant, leaving the other ITR as the original. This result indicated that the ITR modification is repaired in some cases during transfection in HEK293 cells, but not completely, as AAVs containing the BC* ITR are detected.

**Figure 6:**
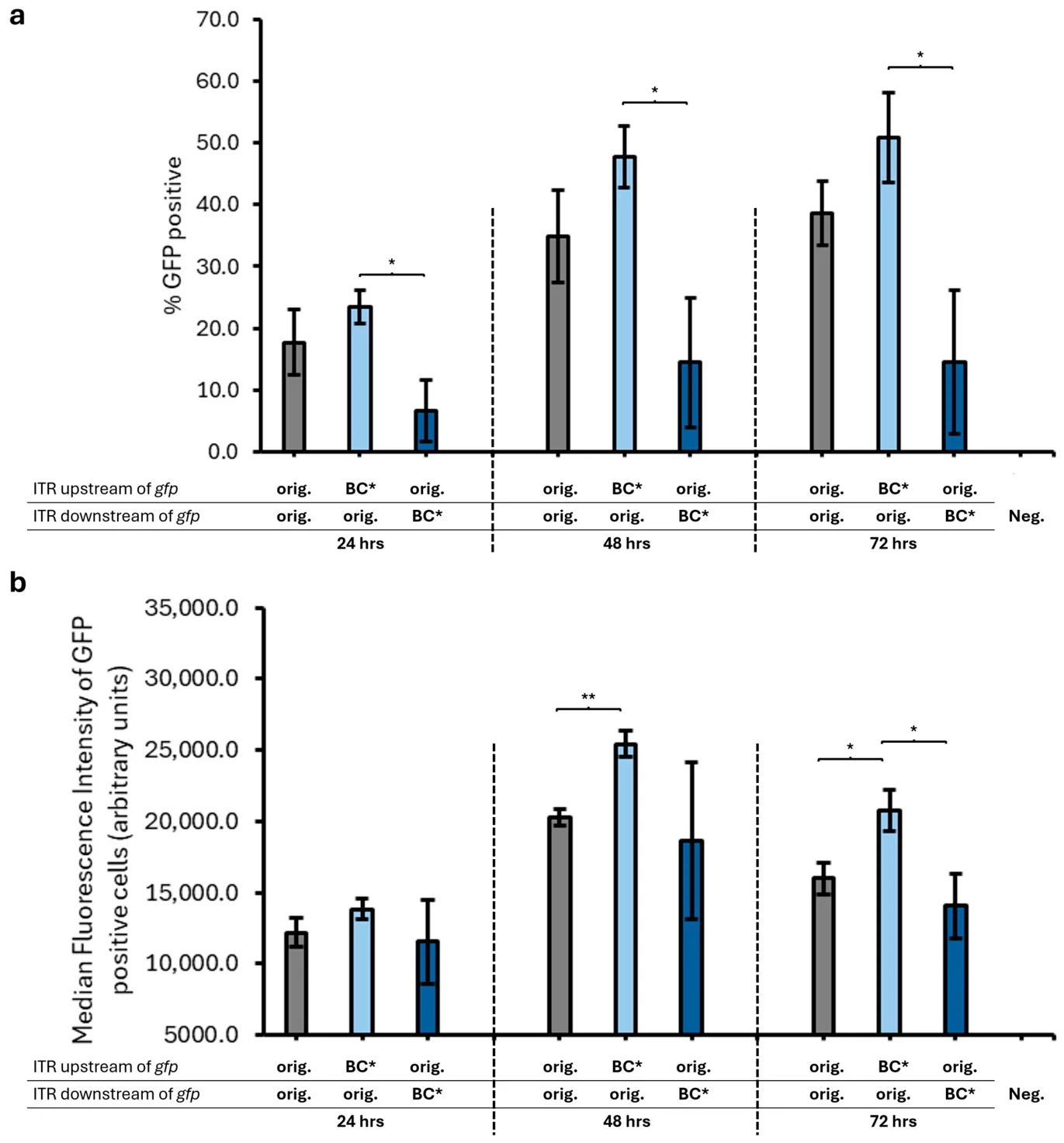
CHO transduction time course using BC* ITR rAAV5 variants (m.o.i. of 10,000 vg/cell). (**a**) percentage of GFP positive cells. (**b**) Median fluorescence intensity of GFP positive cells. Bars represent average of 3 biological replicates. Error bars represent standard deviation. orig., original ITR, Neg., negative control (cells treated with non-AAV control sample). *, *p* < 0.05; **, *p* < 0.01 (*t*-test).

The BC* ITR upstream of *gfp* showed a significantly higher % of GFP+ cells compared to the same variant placed downstream of *gfp* across all time-points (*p* < 0.05, *t*-test; Figure 6a). BC* ITR upstream of *gfp* also showed significantly higher GFP expression compared to the original control at 48 and 72 h (*p* < 0.05, *t*-test, Figure 6b), as well as compared to the BC* ITR downstream of *gfp* sample at 72 h (*p* < 0.05, *t*-test). The results at 24 h were of particular interest: GFP expression was equal across all variants, but there were significantly more GFP+ cells in the BC* ITR placed upstream of *gfp* than when the same variant was placed downstream of *gfp*.

Differences in *gfp* expression could be caused by the ITR variants impacting either second strand synthesis, episome formation or by exerting effects on *gfp* transcription. Given that cells were transduced with equal amounts of viral genomes containing each ITR variant, using identical capsids, it was considered less likely that genome uptake would differ significantly between ITR variants. To address whether effects on second strand synthesis or episome formation caused different amounts of viral genomes to persist in cells, extrachromosomal DNA was extracted from CHO cells 72 h post-transduction with AAV containing either the original ITRs or BC* ITRs placed either upstream or downstream of *gfp*. AAV genomes in extrachromosomal DNA were then quantified by qPCR. Results showed no significant differences in the number of AAV genomes present in extrachromosomal DNA between any of the ITR variants compared to the original (Supplementary Figure S4). This indicated that differences in GFP expression were more likely caused by effects of the BC* ITR on *gfp* transcription rather than ITR variants impacting second strand synthesis or episome formation and thereby causing different amounts of viral genomes to persist in cells.

Interestingly, transfecting ITR-variant pTransgene plasmids into HEK293F cells showed a different pattern than in transduction, as only the ΔBC ITR placed downstream of *gfp* showed significant reductions in the % of GFP+ cells (*p* < 0.01, *t*-test; Supplementary Figure S5a). All other variants showed the same or increased GFP expression and % of GFP+ cells (Supplementary Figure S3). While intriguing, transfection of double-stranded plasmid DNA is not comparable to rAAV transduction. Consequently, this issue was not pursued further.

**Figure 7:**
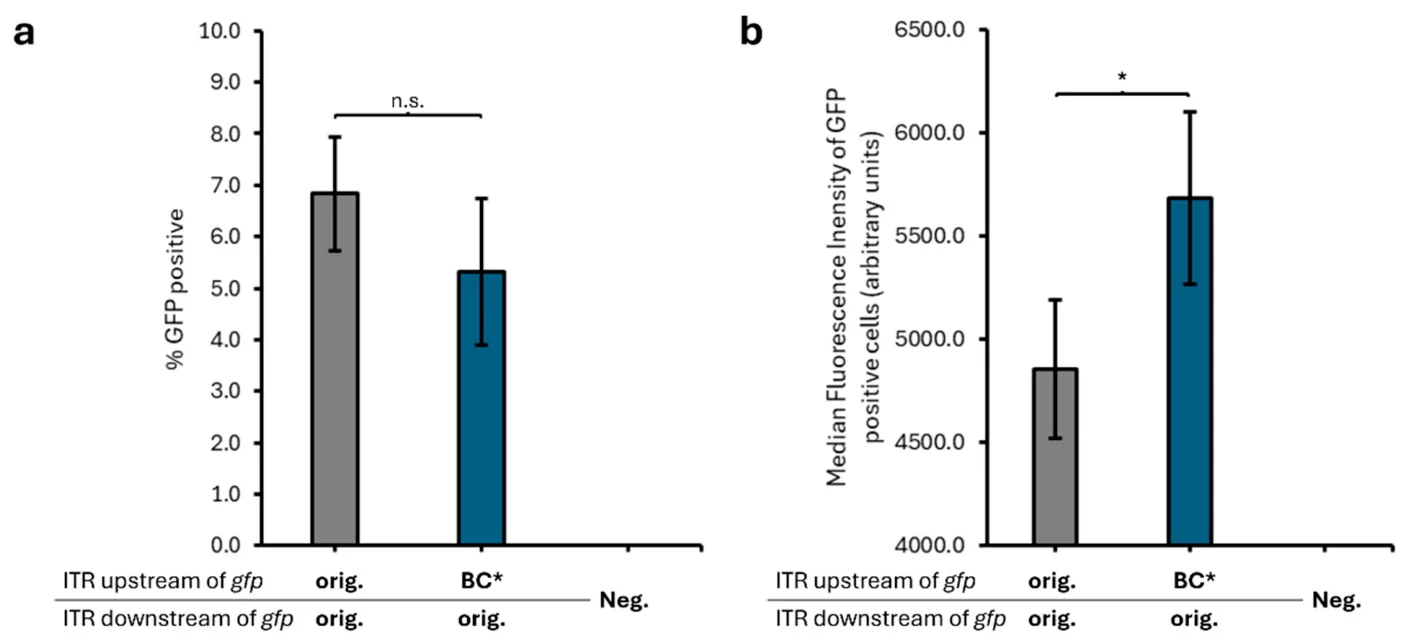
HEK293F cells transduced using rAAV serotype 2 with either original (‘orig.’) or BC* ITR variant upstream of *gfp* (m.o.i. of 1,000 vg/cell). (**a**) Percentage of GFP positive cells. (**b**) Median fluorescence intensity of GFP positive cells. Bars represent an average of 4 biological replicates. Error bars represent standard deviation. Neg., negative control (cells treated with non-AAV control sample). *, *p* < 0.05; n.s., not significant (*t*-test).

To test wider applicability of the observed effect, the original and BC* ITR placed upstream of *gfp* variants were packaged into AAV serotype 2 capsids and transduced into HEK293F cells at an m.o.i. of 1000 vg/cell. Following 72 h incubation, results showed a 17% increase in GFP expression (*p* < 0.05, *t*-test) in the BC* variant upstream of *gfp* (Figure 7b). The % of GFP+ cells was unchanged compared to the original control (Figure 7a). This result demonstrated that the effect of the BC* ITR variant on *gfp* expression can be observed in other cell lines and AAV capsids.

## 3. Discussion

### 3.1. The pUC ori/bleoR Backbone Increased Yields of pHelper but Not pTransgene or pRep/Cap

It was hypothesized that by reducing the size of backbone elements, the proportion of rAAV-relevant DNA would increase; i.e., in a 1 μg/mL of total DNA transfected, the newer plasmids would contain 80.5% rAAV-relevant DNA as opposed to 67.5% for the original material. However, no significant difference in rAAV yield was observed, indicating that rAAV yields are limited by other factors or that if effects are present, they are too small to be significant. The packaging of vector genomes could also be a bottleneck that is unaffected by the changes in plasmid design. This approach could benefit from further deletions within the helper genes, as these had been previously shown to improve rAAV production [33,34].

All triple transfection experiments used a 1:1:1 molar ratio for all plasmids. However, reducing the size of the plasmid backbone changed the molar ratios of the *rep*/*cap* and helper genes. A design-of-experiment approach would be needed to determine whether a different plasmid ratio could improve yields.

The pHelper plasmid yield was increased due to the backbone changes, but the same did not occur for pTransgene and pRep/Cap. We hypothesized that secondary structures could be inhibiting higher plasmid yields specifically for pTransgene and pRep/Cap. As predicted, reducing the strength of ITRs increased plasmid yields for pTransgene. A potentially interesting experiment would be to replace both original ITRs in ColE1 ori/ampR pTransgene with, e.g., the ΔBC ITR variant. This would answer whether the ITRs are the only factor preventing higher pTransgene yields or whether the ori change contributes to higher yields.

### 3.2. ITR Variants Affect Plasmid and rAAV Yield

The effects of ITR variants can be summarized as follows: first, reducing the stability of the hairpins within ITRs increased the plasmid yield. Second, the effect of ITR mutations was cumulative; i.e., adding a second mutant ITR had a stronger effect on plasmid yield than a single ITR variant alone. Third, ITR variants that maintained structural symmetry (BC* and ΔBC) achieved higher plasmid yields than an asymmetrical ITR variant (C*).

Interestingly, placing the ITR modifications downstream of the *gfp* gene did not lead to significant differences in plasmid yield compared to the original control. This may be related to the fact that the pUC ori is placed directly adjacent to the *gfp*-upstream ITR (separated by 208 bp), whereas the downstream ITR is located 749 bp away from pUC ori. Stronger ITR structures placed close to the ori may interfere more with plasmid replication than weaker ITRs or ITRs placed further away from the ori. Previous research indicated that a minimum distance of 438 bp between the ori and the ITR is beneficial for ITR maintenance, as this could relax torsional stress from the plasmid replication bubble [7].

During rAAV production, yields of vector genomes were not impacted by single insertions of ITR variants, either upstream or downstream of the transgene. Significant reductions were only observed when both ITRs were replaced by the C* or ΔBC variants. The latter result matches with previous work in which full deletion of B and C arms (in both ITRs) led to significant reductions in vector genome yields [27].

Similar results were also observed in full deletions of either the B or C arms (or both), which did not cause reductions in vector genome yields when only a single ITR was affected. Furthermore, deletion of either the B, C, or both arms in both ITRs caused significant reductions in vector genome yields [26].

The C* and BC* variants differ from the previously described ITR variants in that they contain only partial deletions of either the B or C arm and maintain the overall T-shaped structure of the ITR. Nevertheless, these variants align with previous results in terms of vector genome yields.

It is likely that ITR deletions would negatively impact secondary structure formation as well as recognition and packaging by Rep. The contrast between the C* and BC* variants points to the role of the secondary structure being particularly important here—while BC* has more deletions and should be less stable, it retains a structure more similar to the original, while the C* structure is uneven. The ΔBC variant cannot form anything close to the ITR structure. As a result, double insertions of C* and ΔBC show significant vector genome yield reductions, which is not the case for BC* double insertions (Figure 4a).

Capsid yields were not impacted by any ITR changes, as expected. In the triple transfection system, *rep* and *cap* are supplied on a separate plasmid under control of their own promoters and are therefore not affected by changes to the ITRs on pTransgene.

### 3.3. ITR Variants Affect Transgene Expression

ITR variants had significant effects on transgene expression following transduction. After endocytosis, trafficking to the nucleus and uncoating, the ITRs flanking the vector genome adopt their secondary structure and initiate second strand synthesis [35]. After double-stranded DNA synthesis has been completed, recombination events between the ITRs lead to the formation of episomes that persist in the nucleus.

Placing the BC* and ΔBC ITR variants upstream of *gfp* increased GFP expression. Previous work identified two cellular proteins in HEK293, ATM and MRN that bind wildtype ITRs and reduce transgene expression [27]. Deletion of the B and C arms led to increased transgene expression but reduced vector genome yields significantly. The same mechanism could be present in CHO cells. Importantly, this work shows that the benefits of this effect can be achieved by replacing only the upstream ITR, without reducing rAAV yield. Interestingly, in work by Chen et al. (2024) [26] full deletions of the B, C, or both arms (in one or both ITRs) did not lead to increased expression of the therapeutic transgene hFIX in transduced Huh7 cells. These discrepancies may be the result of factors specific to the cell lines or transgenes used in these experiments.

Three different mechanisms may be responsible for the effect on transgene expression: first, second strand synthesis could be inhibited by the weaker structure of the ITR variants. Second, recombination events may be reduced between mismatched ITRs. Third, the ITR appears to affect expression of the *gfp* gene but only when it is placed upstream of *gfp*. This final effect is probably due to changes in the recruitment of transcription factors or RNA polymerase. If there were wider effects on, e.g., the entire episome, the effect would be observable in ITR variants placed exclusively downstream of the *gfp* gene, which is not the case.

The ΔBC variant provides an interesting example of the effect of mismatched ITRs: when ΔBC is placed exclusively downstream of *gfp*, expression is lower than the original. However, when both ITRs are replaced with the ΔBC variant, GFP expression returns to the original levels. This indicates that when the ITRs match, episome formation is improved, which enables higher transgene expression.

ITRs also directly influence transgene expression, and this effect could either reinforce or mitigate the effects on second strand synthesis and episome formation. All ITR variants placed exclusively downstream of *gfp* showed significantly lower GFP expression than the original. Conversely, the BC* and ΔBC variants placed exclusively upstream of *gfp* showed significantly higher GFP expression.

In the case of downstream ITR variants, second strand synthesis and episome formation are already inhibited, but the original ITR upstream of *gfp* inhibits transcription, possibly due to interference from ATM and MRN [27]. It is likely that ITR variants upstream of *gfp* are equally impacted by mismatched and weaker ITRs. However, the weaker ITR variants increase transgene expression and compensate for these effects. Future work could quantify the number of vector genomes present in transduced cells in order to test how ITR variants impact second strand synthesis and episome formation.

On a final note, AAVs are known to package backbone DNA, and ITR variants can contribute to this issue [26]. Future work should focus on determining how the BC* ITR variant performs in this regard. Furthermore, the BC* ITR variant should be tested in an in vivo model with a therapeutic transgene.

## 4. Materials and Methods

### 4.1. AAV Plasmids

Plasmids pTransgene, pAAV2/5 and pHelper were purchased from Takara (6650, Takara Bio Europe, Saint-Germain-en-Laye, France). pAAV2/2 was a gift from Melina Fan and the Addgene Research Program (Addgene plasmid #232199; http://n2t.net/addgene:232199 (accessed on 20 April 2023); RRID:Addgene_232199). pUC18T-mini-Tn7T-Zeo-dsRedExpress was a gift from Herbert Schweizer (Addgene plasmid # 65040; http://n2t.net/addgene:65040 (accessed on 20 April 2023); RRID: Addgene_65040) [36]. For pTransgene, the *gfp* gene was inserted into the multi-cloning site downstream of the CMV promoter using EcoRI and SalI restriction sites. Successful insertion was confirmed by nanopore sequencing.

### 4.2. Cell Culture

HEK293F cells were purchased from ThermoFisher (R79007, FisherScientific, Dublin, Ireland) and cultured in BalanCD HEK293 medium (91165, Fujifilm, Tilburg, The Netherlands) supplemented with 4 mM L-glutamine (25030081, FisherScientific, Dublin, Ireland) at 37 °C, 125 rpm, 5% CO_2_. CHO cells were acquired from the National Institute of Standards and Technology (8675, Gaithersburg, MD, USA) [37] and cultured in Ex-Cell CD-CHO Fusion medium (24365C, Merck, Arklow, Ireland) at 37 °C, 125 rpm, 5% CO_2_. Cells were passaged a maximum of 20 times before thawing a new vial.

### 4.3. Plasmid Purification

For minipreps, 2.8 mL of overnight culture in LB (Lennox) media (12780052, FisherScientific, Dublin, Ireland) with appropriate antibiotics was harvested. For maxipreps, 300 mL of LB (Lennox) media (12780052, FisherScientific, Dublin, Ireland) with appropriate antibiotics was harvested. Antibiotic concentration was 100 μg/mL for ampicillin and 50 μg/mL for zeocin. pTransgene strains were cultured at 30 °C, 215 rpm, whereas pRep/Cap and pHelper strains were cultured at 37 °C, 215 rpm. Mini- and maxiprep kits (740588.50 and 740414.50, respectively, Macherey Nagel, Dueren, Germany) were used according to the manufacturer’s instructions. To ensure ITR sequence stability throughout all experiments, a single plasmid maxiprep was performed for each ITR-variant pTransgene and verified by Next Gen sequencing.

### 4.4. PCR

For PCR, Q5 Hotstart polymerase (M0491, New England Biolabs, Ipswich, MA, USA) was used according to the manufacturer’s instructions. For overlap extension PCR, two separate PCRs were performed using primers a + b and c + d. Primers b and c shared a 20 bp complementary region. The amplified DNA was purified using a Nucleospin Gel and PCR clean-up kit (740609.50, Macherey Nagel, Dueren, Germany) and mixed at a 1:1 molar ratio in a total amount of 100 ng. This mixture was used as the template in a subsequent PCR in which the incorporated overlap regions of primers b and c hybridize and enable amplification without primers. For primer sequences, see Supplementary Table S2. This reaction was performed for 15 cycles (using an annealing temperature based on the overlap region) before adding primers a and d to the mixture, then continuing the reaction for a further 30 cycles (annealing temperature was adjusted based on primers a and d).

### 4.5. Plasmid Cloning

For DNA restriction, up to 1 μg of DNA was added to nuclease-free water with 2 μL of FastDigest buffer, as well as EcoRI, SgfI, SalI or PacI FastDigest enzymes (FD0274, FD2094, FD0644, FD2204, respectively, FisherScientific, Dublin, Ireland) up to a 20 μL total volume. For vector fragments, 1 μL of FastAP alkaline phosphatase (EF0654, FisherScientific, Dublin, Ireland) was added to the reaction. Incubation was performed for 20 min at 37 °C, followed by an 80 °C, 5 min inactivation step.

Ligations were performed using a 3:1 insert:vector molar ratio with T4 ligase (EL0014, FisherScientific, Dublin, Ireland) at 16 °C overnight. In total, 2 μL of ligation mix was transformed into 50 μL of NEB stbl *E. coli* (C3040H, New England Biolabs, Ipswich, MA, USA) according to the manufacturer’s instructions. Lennox agar selection plates contained either 100 μg/mL ampicillin or 50 μg/mL zeocin. pTransgene strains were cultured at 30 °C, whereas pRep/Cap and pHelper strains were cultured at 37 °C.

### 4.6. Sequencing

All sequencing was performed by Eurofins whole plasmid nanopore sequencing service (Dublin, Ireland).

### 4.7. Transfection

HEK293F cells were seeded in BalanCD media with 4 mM L-glutamine at 1.11 × 10^6^ cells/mL in a total volume of 1.8 mL in 6-well plates (150239, FisherScientific, Dublin, Ireland), then incubated at 37 °C, 125 rpm, 5% CO_2_ for 24 h. For triple transfection, pTransgene, pRep/Cap and pHelper were mixed in a 1:1:1 molar ratio to a total amount of 2 μg DNA in 0.1 mL of BalanCD + L-glutamine. For single plasmid transfections, 2 μg of the relevant plasmid was added to the same media. For negative controls used in qPCR and transduction experiments, 0.38 μg of only the pTransgene plasmid was transfected. PEI was prepared at a 1:2 ratio, i.e., 4 μL for 2 μg of DNA in 0.1 mL BalanCD + L-glutamine. The PEI mix was added to the DNA mix, vortexed for 2 s at high speed and incubated at room temperature for 15 min, then added to the cells. Cells were incubated for a further 72 h at 37 °C, 125 rpm, 5% CO_2_.

### 4.8. AAV Harvesting and Purification

A total of 500 μL of transfected cells (including negative controls) was moved to a new 6-well plate and mixed with 500 μL of fresh BalanCD + L-glutamine. MgCl_2_ (AM9530G, FisherScientific, Dublin, Ireland) and Benzonase (1016540001, Merck) were added to a final concentration of 2 mM and 100 U/mL, respectively. Incubation continued for 1 h at 37 °C, 125 rpm, 5% CO_2_. Cells were transferred to Eppendorf tubes, centrifuged at 16,000 *g* for 20 min at 4 °C. The supernatant was removed and stored at −80 °C. Cell pellets were resuspended in 1 mL of fresh media, sonicated for 2 min at 100% amplitude and transferred to 6-well plates. MgCl_2_ and Benzonase were added again to a final concentration of 2 mM and 100 U/mL, respectively, followed by incubation for a further hour at 37 °C, 125 rpm, 5% CO_2_. These samples were moved to Eppendorf tubes, centrifuged at 16,000 *g* for 20 min at 4 °C. The supernatants were stored at −80 °C.

### 4.9. qPCR

Vector genome titers were measured for cell pellet and supernatant samples. Initially, 10 μL of sample was mixed with 80 μL of nuclease-free water containing 100 U of Benzonase (final concentration 10U/mL). Following incubation at 37 °C, 30 min, 10 μL of 0.5 M EDTA was added. Samples were further incubated at 68 °C, 10 min to inactivate Benzonase. In total, 10 μL of these samples was added to 70 μL of Proteinase K solution in nuclease-free water (6 U/mL final concentration) and incubated at 50 °C for 1 h, then 95 °C for 20 min. The resulting sample has been 80-fold diluted.

qPCR was performed using Fast SYBR Green Master Mix (4385618, FisherScientific, Dublin, Ireland) and primers specific to the AAV2 ITRs [38] on a QuantStudio 5 Real-Time PCR System (FisherScientific, Dublin, Ireland). A standard curve ranging from 4 × 10^3^ copies to 4 × 10^8^ copies was generated using a PCR product derived from the ITR region. Only results within this standard range were considered valid. In control samples (i.e., cells transfected with only pTransgene), results were lower than 4 × 10^3^ copies, which indicated successful removal of plasmid DNA. A rAAV2 reference standard was employed to standardize results across different qPCR experiments (VR1616, LGC standards, Teddington, UK). A 2-step standard protocol was used, consisting of 50 °C for 2 min, 95 °C for 10 min, followed by 45 cycles of 95 °C for 15 s and 60 °C for 1 min. A melt curve was included at the end (95 °C for 15 s, then 60 °C for 1 min and a continuous ramp from 60° to 95 °C at 0.15 °C per second). The threshold was manually set to 0.4. Vector genome titers were calculated using QuantStudio design and analysis software (version 1.5.1) based on the standard curve. Negative controls (nuclease-free water as template) were included. Primer efficiency ranged between 90 and 110%. R^2^ values ranged above 0.980. Primer sequences are shown in Supplementary Table S3.

### 4.10. ELISA

The Progen AAV5 ELISA kit (PRAAV5, Progen, Heidelberg, Germany) was used according to the manufacturer’s instructions. Results were read on a Varioskan LUX plate reader (FisherScientific, Dublin, Ireland) at 450 and 650 nm.

### 4.11. Transduction

CHO cells were seeded at 10,000 cells per well in a 24-well cell culture-treated plate in 500 μL DMEM media (11960044, FisherScientific, Dublin, Ireland) supplemented with 10% fetal bovine serum (10082147, Gibco, Grand Island, NY, USA) and 2mM L-glutamine. Cells were cultured overnight at 37 °C, 5% CO_2_.

For AAV2/2 transduction experiments, HEK293F cells were adapted to adherent mode through culturing in MEM (10370047, Gibco) supplemented with 2mM L-glutamine, 10% FBS and use of tissue culture-treated T-flasks. Cells were seeded at 10,000 cells per well in a 24-well plate in 500 μL complete MEM medium. Cells were cultured overnight at 37 °C, 5% CO_2_.

AAV samples were diluted in DMEM to a concentration of 2 × 10^8^ vg/mL in a total volume of 1.65 mL. A total of 0.5 mL of diluted AAVs was then added to individual wells, resulting in an m.o.i. of 10,000 vg/cell. Each transduction was performed in biological triplicates. For negative controls, 0.5 mL of the negative control sample (pTransgene-only transfection, see transfection methods description) was added to cells. Cells were incubated for a further 72 h, resuspended and analyzed by flow cytometry.

### 4.12. Extrachromosomal DNA Purification

Extrachromosomal DNA was purified following the method described by [39]. At 72 h post-transduction, cells were dispersed with trypsin and washed three times with Mg^2+^/Ca^2+^ free phosphate-buffered saline, resuspended in 250 μL 50 mM Tris-HCl, pH 7.5, 10 mM EDTA containing 100mg/mL RNase A and lysed by adding 250 μL 1.2% sodium dodecyl sulfate (SDS). The suspension was gently mixed by inverting, then incubated for 5 min at room temperature. Next, 350 μL 3 M CsCl, 1 M potassium acetate and 0.67 M acetic acid was added to precipitate chromosomal DNA and cellular debris. Tubes were gently mixed and placed on ice for 15 min, then centrifuged for 15 min at 14,000 *g*. The supernatant was loaded onto a silica gel membrane column (740588.50, Macherey Nagel, Dueren, Germany) followed by washing and elution in 20 μL TE buffer (10 mM Tris-HCl, pH 8.0, 1 mM EDTA) according to manufacturer’s instructions.

### 4.13. Flow Cytometry

Flow cytometry was performed using a Cytoflex instrument (Beckman-Coulter, Amersham, UK). Results were analyzed using Floreada software (beta version) (https://floreada.io (accessed on 19 May 2025)). For gating strategy, see Supplementary Figure S6.

### 4.14. Statistical Analysis

Statistical significance was determined using Excel’s unpaired two-tailed *t*-test. *p* values below 0.05 were considered significant.

## 5. Conclusions

In summary, modification of the plasmid backbone led to a 140% increase in pHelper plasmid yield. Furthermore, placing the BC* ITR variant upstream of the transgene resulted in a 57% increase in pTransgene plasmid yield and a 28% increase in GFP expression, without reducing rAAV yields or affecting packaging efficiency. As the AAV serotype 2 ITR is used widely across the industry, these modifications offer a promising avenue for reducing rAAV production costs and increasing transgene expression during gene therapy.

## Figures and Tables

**Table 1:** Plasmid miniprep yields of ITR variants in pTransgene compared to original ITR control. Three biological replicates per sample. *, *p* < 0.05; **, *p* < 0.01; n.s., not significant (*t*-test).

ITR Variant and Position		Plasmid Yield Change vs. Original	*p*-Value
Upstream of *gfp*	Downstream of *gfp*		
original	original	n/a	n/a
C*	original	0%	n.s.
BC*	original	+20%	*
ΔBC	original	+16%	**
original	C*	−3%	n.s.
original	BC*	−5%	n.s.
original	ΔBC	−5%	n.s.
C*	C*	+12%	*
BC*	BC*	+35%	**
ΔBC	ΔBC	+35%	**

## Data Availability

All data associated with this study are present in the paper or the supplemental information.

## Supplementary Materials

The following supporting information can be downloaded at: https://www.mdpi.com/article/10.3390/ijms27177603/s1.
