# Supplementary material for: Modified Plasmids and Inverted Terminal Repeats Enhance Adeno-Associated Virus Production and Performance
Source: Int J Mol Sci. 2026 Aug 25;27(17):7603. doi: 10.3390/ijms27177603 (PMC13565880; doi:10.3390/ijms27177603)
Supplement: Supplementary file 1 [file ijms-27-07603-s001.zip › ijms-4357192-supplementary.pdf]

Supplemental data

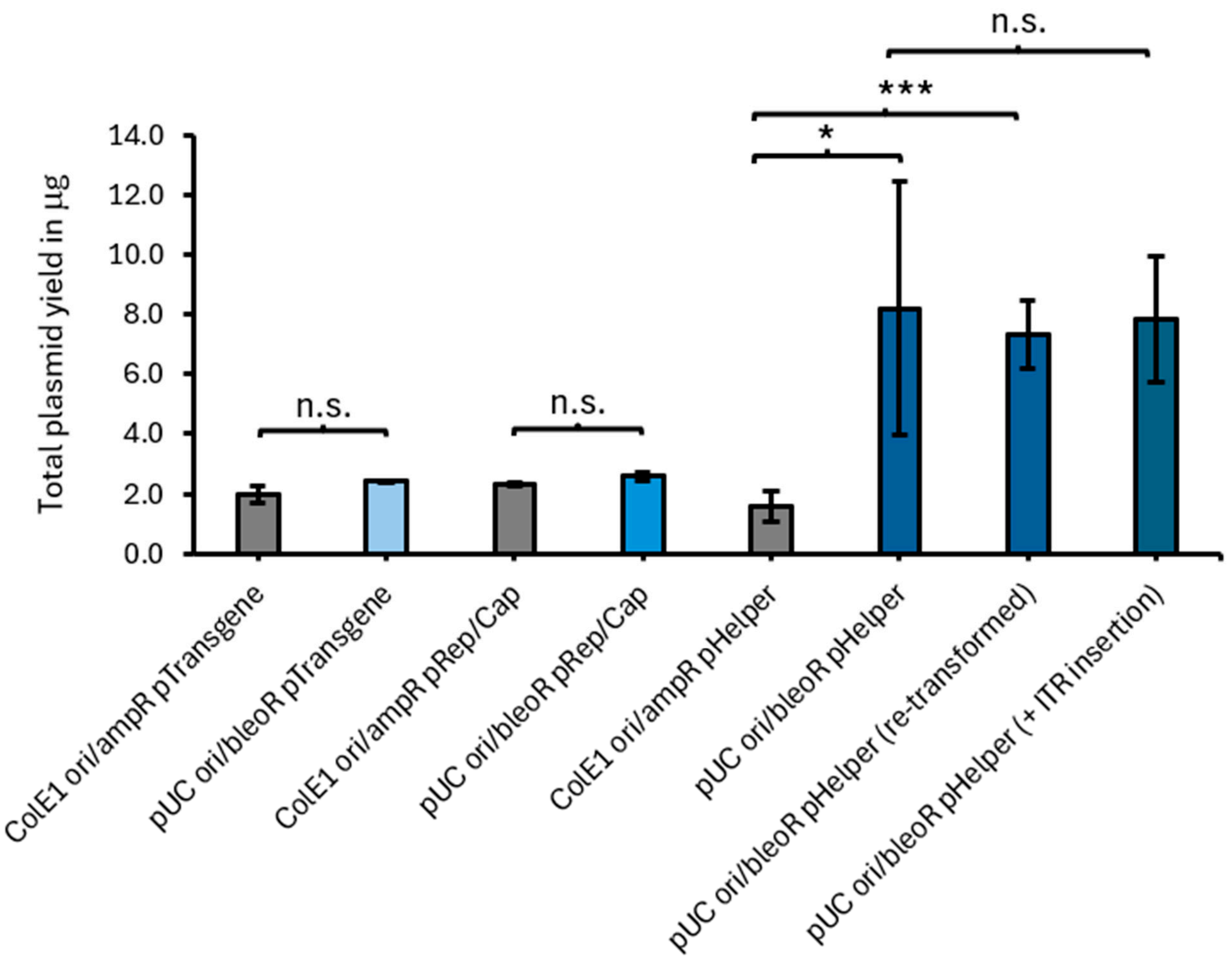

Sup. Figure S1: Miniprep yields of original and pUC ori/bleoR plasmids. Bars represent average of 3 biological replicates. Error bars represent standard deviation. *, $p < 0.05$; ***, $p < 0.001$, n.s., not significant (*t*-test).

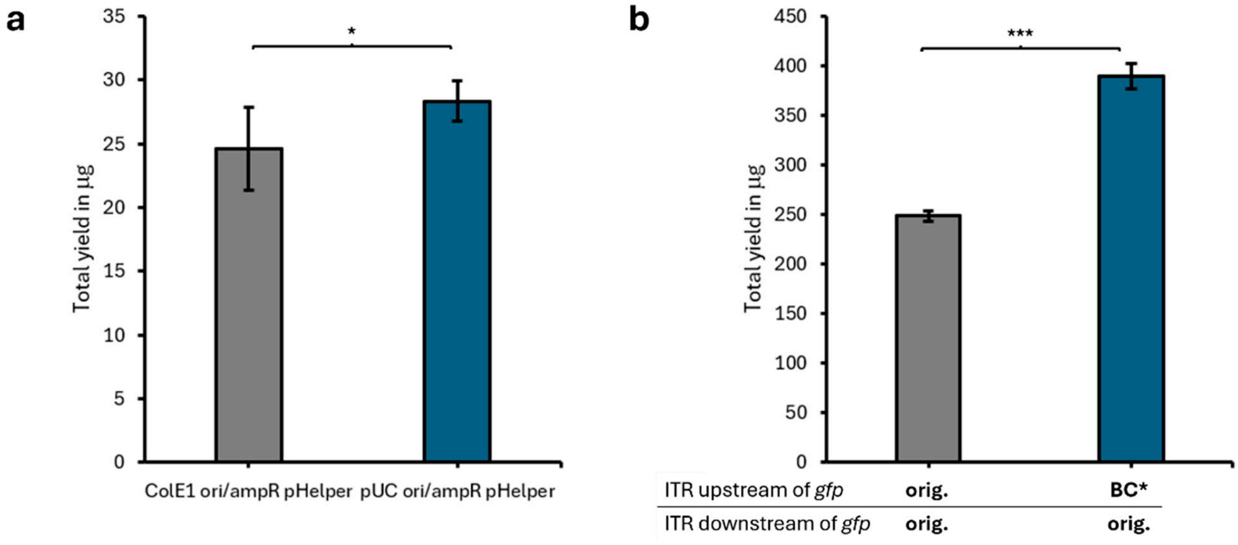

Sup. Figure S2: Plasmid yields for pHelper and pTransgene variants. **a,** miniprep yields for ColE1 ori/ampR and pUC ori/ampR pHelper. **b,** maxiprep yields pTransgene with original ('orig.') or BC* ITR inserted upstream of *gfp*. Bars represent average of 3 biological replicates. Error bars represent standard deviation. *, p < 0.05, ***, p < 0.001 (t-test).

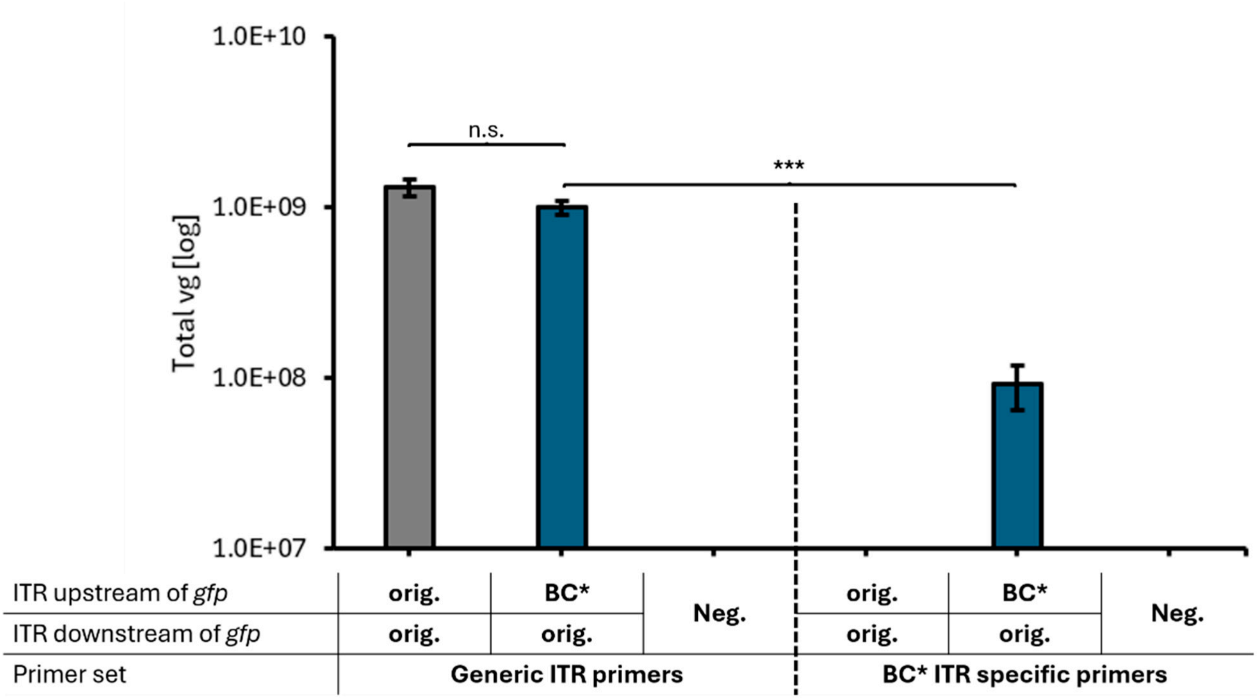

Sup. Figure S3: Genome titers for rAAV5 (produced from 500 µL HEK293 culture) using original ('orig.') and BC* ITR-variant plasmids. Total viral genomes were quantified by qPCR using generic ITR primers or BC* specific ITR primers. Bars represent average of 4 biological replicates. Error bars represent standard deviation. Neg., negative control (cells transfected with pTransgene only). ***, p < 0.001; n.s., not significant (t-test).

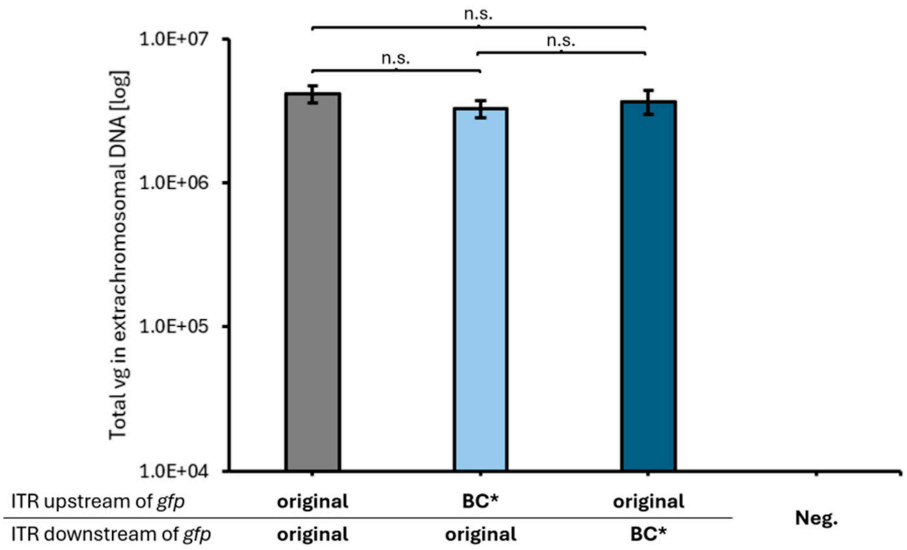

Sup. Figure S4: Total viral genomes (vg) present in extrachromosomal DNA extracted from CHO cells 72 hrs post-transduction with AAVs containing either original ITRs, or BC* ITRs placed upstream or downstream of *gfp*. Bars represent average of 4 biological replicates. Error bars represent standard deviation. Neg., negative control: non-AAV transduced CHO cell sample. n.s., not significant (p > 0.05, t-test).

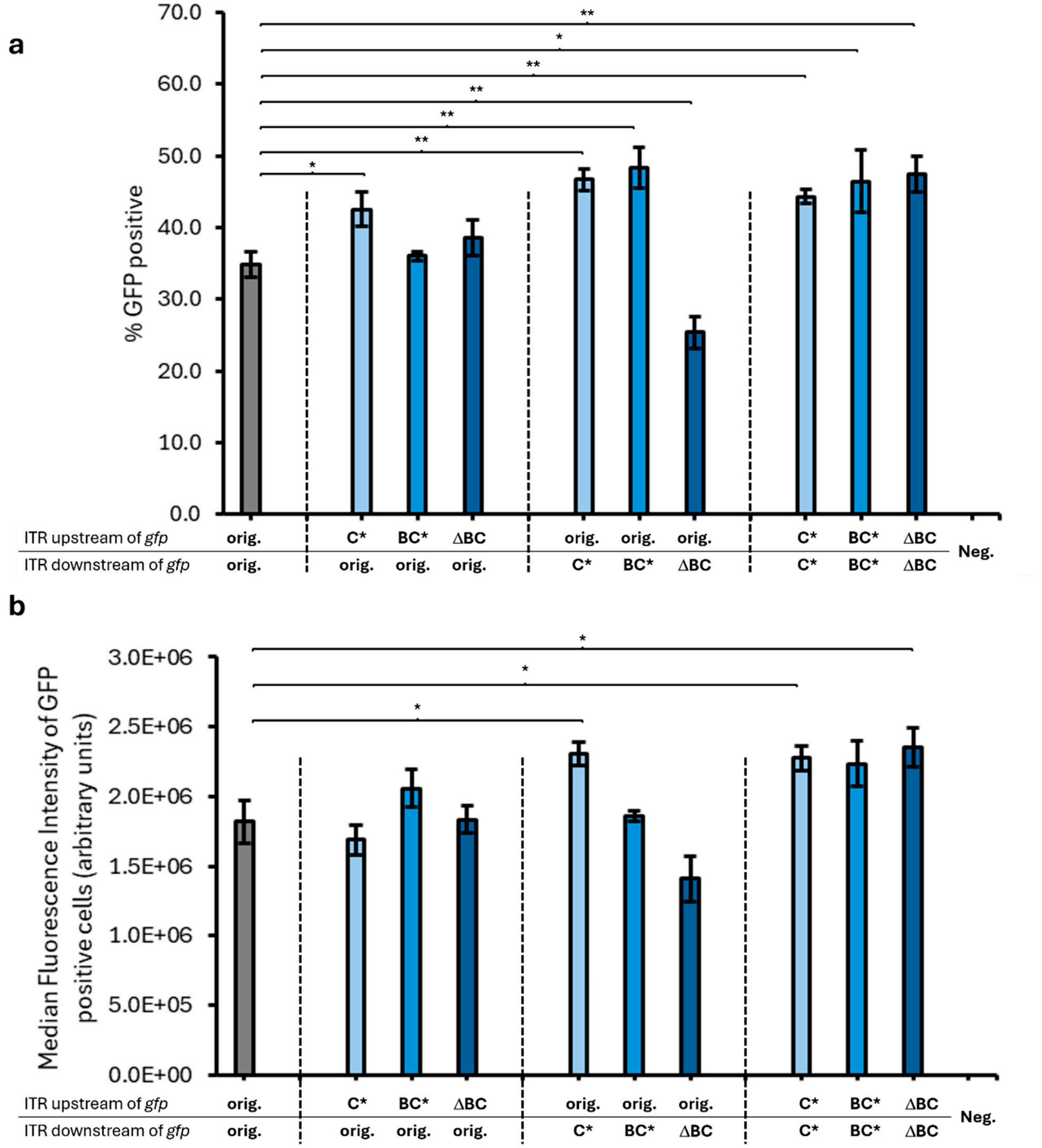

Sup. Figure S5: Transfection of HEK293F cells with 2 µg/mL of pTransgene ITR variants. **a,** percentage of GFP positive cells. **b,** Median fluorescence intensity. Bars represent average of 3 biological replicates. Error bars represent standard deviation. *, $p < 0.05$; **, $p < 0.01$ (*t*-test).

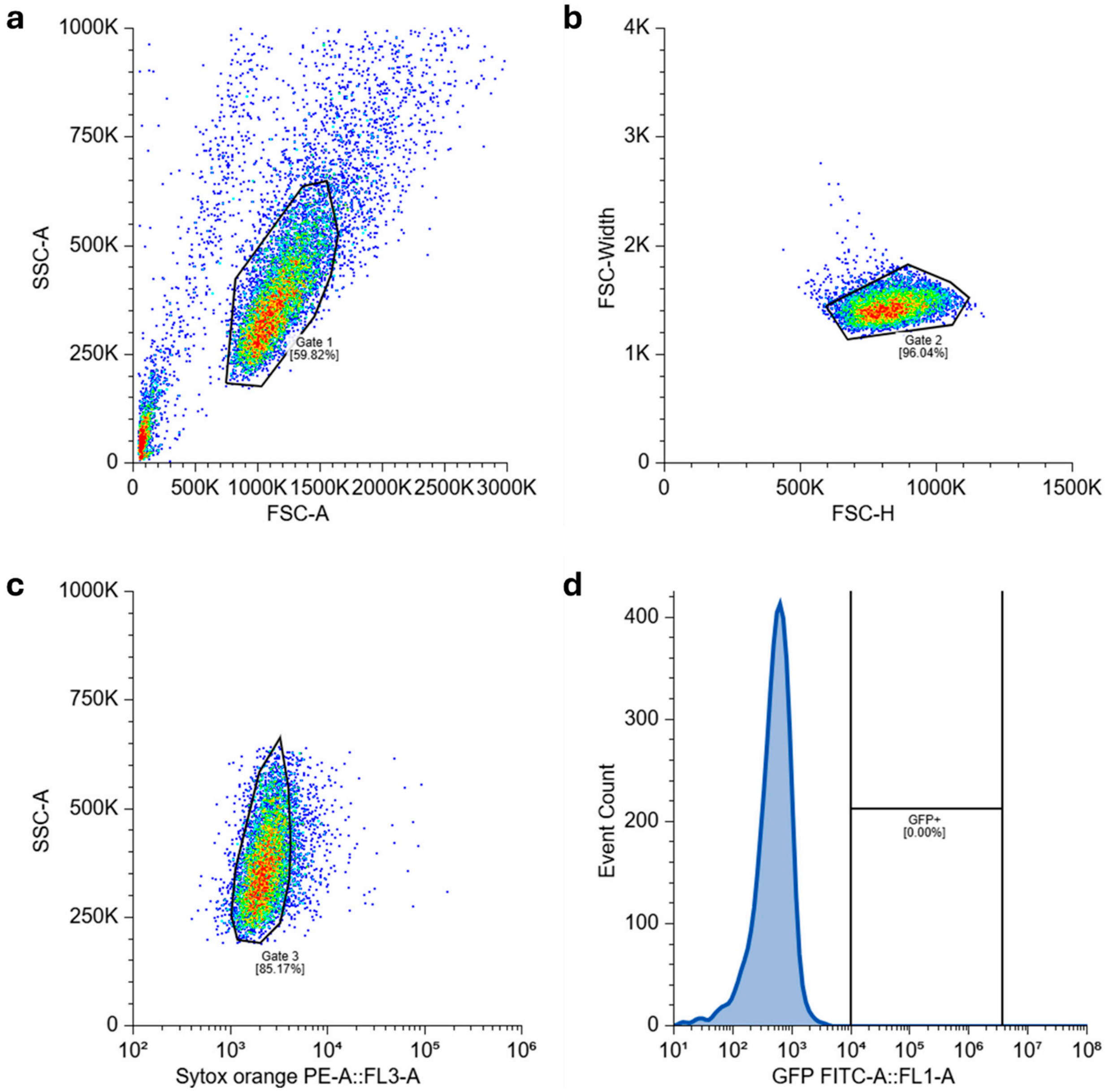

Sup. Figure S6: Gating strategy for flow cytometry showing negative control (cells treated with non-AAV sample). **a,** cells were gated to remove debris. **b,** doublet exclusion. **c,** live/dead gating using Sytox orange. **d,** fluorescent marker gating (GFP). Plots were produced using Floreada software.

Sup. Table S1. Detailed sequence of original ITR and ITR variants.

ITR variant	Sequence (5' → 3')	Size (bp)
Original AAV2-derived ITR sequence	CTGCGCGCTCGCTCGCTCACTGAGGCCGCCCGGGCA AAGCCCGGGCGTCGGGCGACCTTTGGTCGCCCGGCC TCAGTGAGCGAGCGAGCGCGCAGAGAGGGAGTGGCC AACTCCATCACTAGGGGTTCCT	130
C* ITR	CTGCGCGCTCGCTCGCTCACTGAGGCCGCAAAGCGTC GGGCGACCTTTGGTCGCCCGGCCTCAGTGAGCGAGC GAGCGCGCAGAGAGGGAGTGGCCAACTCCATCACTAG GGGTTCCT	118
BC* ITR	CTGCGCGCTCGCTCGCTCACTGAGGCCGCCCAAAGG GCGTCGGTTTCCGGCCTCAGTGAGCGAGCGAGCGCG CAGAGAGGGAGTGGCCAACTCCATCACTAGGGGTTCCT	110
ΔBC ITR	CTGCGCGCTCGCTCGCTCACTGAGGCTTTGCCTCAGTG AGCGAGCGAGCGCGCAGAGAGGGAGTGGCCAACTCC ATCACTAGGGGTTCCT	90

Sup. Table S2. Primer sequences used for overlap PCRs

Description	Overlap PCR function	Sequence (5' → 3')
C* overlap primer 1	**b**	CGACGCTTTGCGGCCTCAGTGAGCGAGC
C* overlap primer 2	**c**	GGCCGCAAAGCGTCGGGCGACCTTTGG
BC* overlap primer 1	**b**	GGAAACCGACGCCCTTTGGGCGGCCTCAGTGAGC
BC* overlap primer 2	**c**	CCAAAGGGCGTCGGTTTCCGGCCTCAGTGAGCGAG
DBC overlap primer 1	**b**	TCACTGAGGCAAAGCCTCAGTGAGCGAGCGAG
DBC overlap primer 2	**c**	TCACTGAGGCTTTGCCTCAGTGAGCGAGCGAG
PacI site primer (for upstream of *gfp* ITRs)	**a**	CATCCCCCTTTCGCCAGCTGGTTAATTAATG

EcoRI site primer (for upstream of *gfp* ITRs)	**d**	CGCGGAATTCTCGCGAATCCCAATTC
SgfI site primer (for downstream of *gfp* ITRs)	**a**	TATAATGCGATCGCAGTATACCTTAAGGAATCCCCTGATAAC
EcoRI site primer (for downstream of *gfp* ITRs)	**d**	TCGCGAGAATTCCGCGGG

Sup. Table S3. qPCR primer sequences

**Description**	**Sequence (5' → 3')**
ITR forward qPCR primer	GGAACCCCTAGTGATGGAGTT
ITR reverse qPCR primer	CGGCCTCAGTGAGCGA
BC* ITR reverse qPCR primer	CCAAAGGGCGTCGGTTTCC
